# Supplementary material for: Deep learning framework for sensor array precision and accuracy enhancement
Source: Sci Rep. 2023 Jul 11;13:11237. doi: 10.1038/s41598-023-38290-8 (PMC10336090; doi:10.1038/s41598-023-38290-8)
Supplement: Supplementary file 1 — Supplementary Information. [file 41598_2023_38290_MOESM1_ESM.pdf]

Deep learning framework for sensor array precision and accuracy enhancement : Supplementary Information

Julie Payette[1], Fabrice Vaussenat[1], and Sylvain Cloutier[1,*]

[1]École de technologie supérieure, Department of Electrical Engineering, Montréal, H3C 1K3, Canada
[*]SylvainG.Cloutier@etsmtl.ca

ABSTRACT

In the upcoming years, artificial intelligence (AI) is going to transform the practice of medicine in most of its specialties. Deep learning can help achieve better and earlier problem detection, while reducing errors on diagnosis. By feeding a deep neural network (DNN) with the data from a low-cost and low-accuracy sensor array, we demonstrate that it becomes possible to significantly improve the measurements' precision and accuracy. The data collection is done with an array composed of 32 temperature sensors, including 16 analog and 16 digital sensors. All sensors have accuracies between 0.5-2.0°C. 800 vectors are extracted, covering a range from to 30 to 45°C. In order to improve the temperature readings, we use machine learning to perform a linear regression analysis through a DNN. In an attempt to minimize the model's complexity in order to eventually run inferences locally, the network with the best results involves only three layers using the hyperbolic tangent activation function and the Adam Stochastic Gradient Descent (SGD) optimizer. The model is trained with a randomly-selected dataset using 640 vectors (80% of the data) and tested with 160 vectors (20%). Using the mean squared error as a loss function between the data and the model's prediction, we achieve a loss of only 1.47x10^{-4} on the training set and 1.22x10^{-4} on the test set. As such, we believe this appealing approach offers a new pathway towards significantly better datasets using readily-available ultra low-cost sensors.

Materials

Sensors

The DS18B20 IC-based sensor communicates over a 1-Wire bus, which implies it only needs one data line for communication with the microprocessor. Its operating range is between -55°C and 125°C. Its accuracy varies between 0.5-2.0°C, the best obtained for temperatures in a -10 to 85°C range. The NXRT15WF104FA1B040 sensor is a NTC thermistor with a 100kΩ resistance at 25°C. Its operating range is between -40°C and 125°C. Its precision varies but stays around 2.0°C in this range.

Methods

Data set

The training set was randomly selected but manually verified to make sure the whole temperature range was covered. Below is the distribution for our training set.

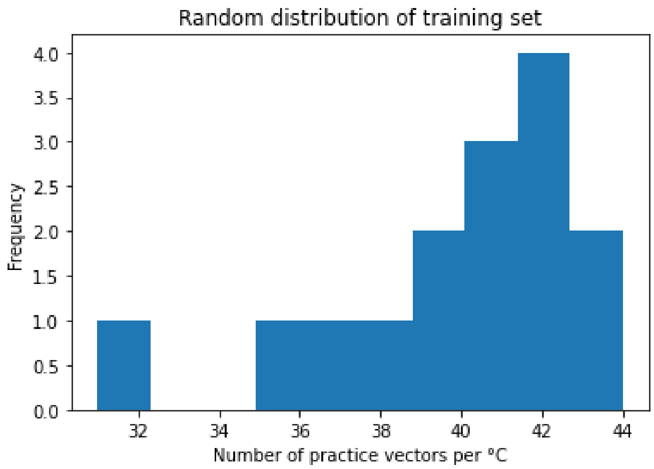

Figure S1. Random distribution of practice vectors composing our training set, with a mean of 40 vectors per ° C, as targeted.

Neural Network

As mentionned in the paper, we tried different parameters for our model in order to get an optimized version. It was clear that the hyperbolic tangent activation function for both layers worked the best. As for the number of epochs, as depicted by Figure 5 in text, the loss is already stable after 100 epochs. Therefore, adding epochs only results in overfitting after approximately 320 epochs. For 600 epochs, we get a 3.47x10^{-2} loss on the test set, which is roughly a factor two hundred (200$\times$) larger than our best MSE result. The step in figure below shows the overfitting.

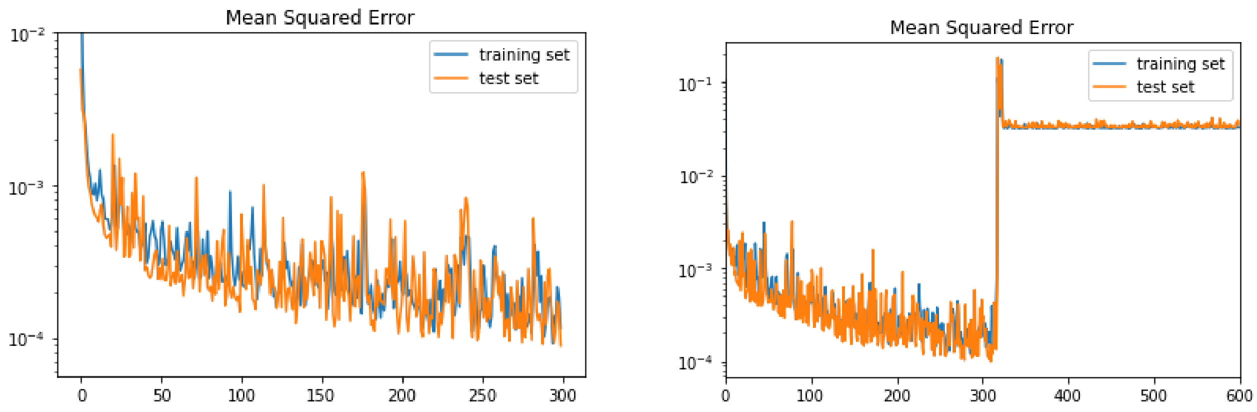

(a) (b)

Figure S2. (a) MSE loss function on logarithmic scale of our model (b) MSE loss function on logarithmic scale of our model for 600 epochs

Furthermore, we tried adding an extra hidden layer to our model to study the effect. Adding a 12-neuron layer resulted in a 2.27x10^{-4} loss for the test set, which is roughly twice larger than our best MSE result.

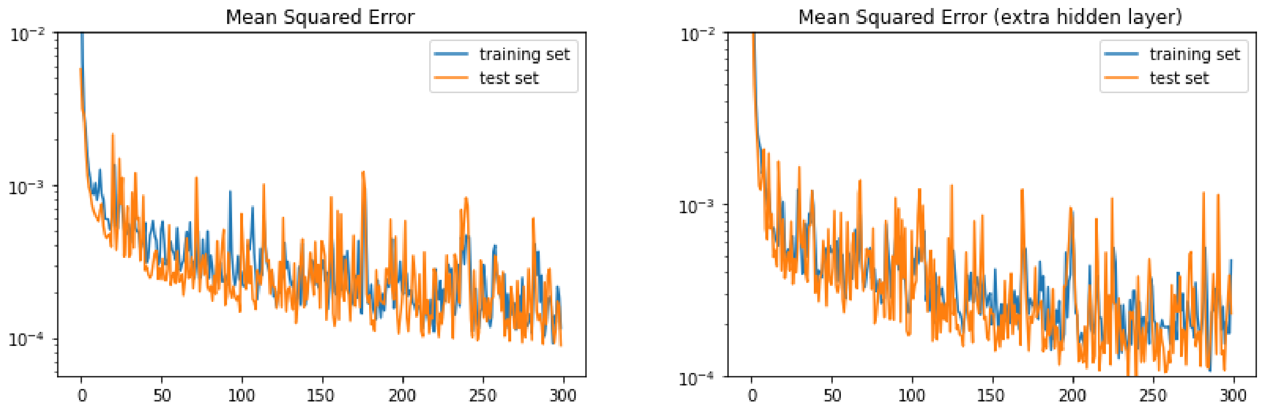

(a) (b)

Figure S3. (a) MSE loss function on logarithmic scale of our model (b) MSE loss function on logarithmic scale of our model with extra hidden layer

In order to optimize the hyperparameters of the model, we used Python's Scikit-Learn module to perform a five-cross validation grid search. We tested learning rate, number of epochs, activation functions, model optimizer and number of neurons in hidden layer. All the optimal choices are in bold in Table S1 below and were chosen as the parameters' values for our model.

Learning rate	MSE	Activation	MSE	Optimizer	MSE
0.001	0.000217±0.000018	softmax	0.195358±0.005422	SGD	0.001427±0.000364
**0.01**	**0.000157±0.000016**	**tanh**	**0.000320±0.000055**	Adam	0.000760±0.000401
0.1	0.195358±0.005422	sigmoid	0.000360±0.000049	Adagrad	0.002351±0.000127
0.2	0.987011±1.120184	ReLU	0.259489±0.183432	Adadelta	0.020287±0.006209
0.3	0.195358±0.005422	linear	0.000423±0.000188	**Adam**	**0.000200±0.000036**
				Adamax	0.000382±0.000122
Epochs	MSE	Neuron number	MSE		
100	0.000682±0.000241	5	0.000297±0.000109		
200	0.000468±0.000088	10	0.000294±0.000073		
250	0.000325±0.000093	15	0.000352±0.000164		
**300**	**0.000213±0.000037**	**20**	**0.000246±0.000035**		
350	0.000523±0.000196	25	0.000307±0.000056		
400	0.000281±0.000093	30	0.000288±0.000042		

Table S1. Hyperparameters tuning and options for our deep learning model. Best selection is in bold, according to lowest MSE.

Results

For the paper, we trained the model with both types of sensors jointly. Here are the prediction results for the same model, but trained only with the digital readings (yellow) and the analog readings (blue). Their respective prediction are also on the graph.

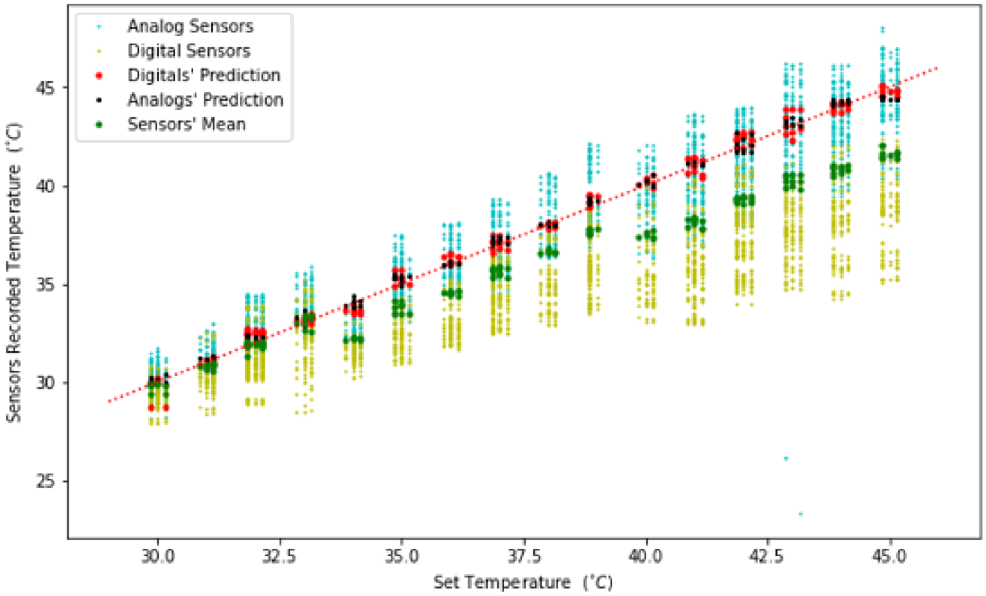

Figure S4. Independent model's prediction according to temperature sensors' type
